# Blocking of efflux transporters in rats improves translational validation of brain radioligands

**DOI:** 10.1186/s13550-020-00718-x

**Published:** 2020-10-19

**Authors:** Vladimir Shalgunov, Mengfei Xiong, Elina T. L’Estrade, Nakul R. Raval, Ida V. Andersen, Fraser G. Edgar, Nikolaj R. Speth, Simone L. Baerentzen, Hanne D. Hansen, Lene L. Donovan, Arafat Nasser, Siv T. Peitersen, Andreas Kjaer, Gitte M. Knudsen, Stina Syvänen, Mikael Palner, Matthias M. Herth

**Affiliations:** 1 Department of Drug Design and Pharmacology, Faculty of Health and Medical Sciences, University of Copenhagen, Jagtvej 160, 2100 Copenhagen, Denmark; 2 Department of Clinical Physiology, Nuclear Medicine and PET, Copenhagen University Hospital, Rigshospitalet, Blegdamsvej 9, 2100 Copenhagen, Denmark; 3 Neurobiology Research Unit, Rigshospitalet, Blegdamsvej 9, 2100 Copenhagen, Denmark; 4 Department of Public Health and Caring Sciences/Geriatrics, Rudbeck Laboratory, Uppsala University, 75185 Uppsala, Sweden; 5 Radiation Physics, Nuclear Medicine Physics Unit, Skånes University Hospital, Barngatan 3, 222 42 Lund, Sweden; 6 A. A. Martinos Center for Biomedical Imaging, Massachusetts General Hospital, 149 13th Street, Charlestown, MA 02129 USA; 7 Cluster for Molecular Imaging, Department of Biomedical Sciences, Faculty of Health and Medical Sciences, University of Copenhagen, Blegdamsvej 3, 2200 Copenhagen, Denmark; 8 Center for Translational Neuromedicine, Faculty of Health and Medical Sciences, University of Copenhagen, Blegdamsvej 3B, 2200 Copenhagen, Denmark; 9 Institute of Clinical Medicine, University of Copenhagen, Blegdamsvej 3B, 2200 Copenhagen, Denmark

**Keywords:** P-gp, Efflux transporter, PET, Rodents, Pigs, Rats, Translation

## Abstract

**Background:**

Positron emission tomography (PET) is a molecular imaging technique that can be used to investigate the in vivo pharmacology of drugs. Initial preclinical evaluation of PET tracers is often conducted in rodents due to the accessibility of disease models as well as economic considerations. Compared to larger species, rodents display a higher expression and/or activity of efflux transporters such as the P-glycoprotein (P-gp). Low brain uptake could, therefore, be species-specific and uptake in rodents not be predictive for that in humans. We hypothesized that a better prediction from rodent data could be achieved when a tracer is evaluated under P-gp inhibition. Consequently, we compared the performance of eight neuroreceptor tracers in rats with and without P-gp inhibition including a specific binding blockade. This data set was then used to predict the binding of these eight tracers in pigs.

**Methods:**

PET tracers targeting serotonin 5-HT_2A_ receptors ([^18^F]MH.MZ, [^18^F]Altanserin, [^11^C]Cimbi-36, [^11^C]Pimavanserin), serotonin 5-HT_7_ receptors ([^11^C]Cimbi-701, [^11^C]Cimbi-717 and [^11^C]BA-10) and dopamine D_2/3_ receptors ([^18^F]Fallypride) were used in the study. The brain uptake and target-specific binding of these PET radiotracers were evaluated in rats with and without inhibition of P-gp. Rat data were subsequently compared to the results obtained in pigs.

**Results:**

Without P-gp inhibition, the amount of target-specific binding in the rat brain was sufficient to justify further translation for three out of eight evaluated tracers. With P-gp inhibition, results for five out of eight tracers justified further translation. The performance in pigs could correctly be predicted for six out of eight tracers when rat data obtained under P-gp inhibition were used, compared to four out of eight tracers without P-gp inhibition.

**Conclusions:**

P-gp strongly affects the uptake of PET tracers in rodents, but false prediction outcomes can be reduced by evaluating a tracer under P-gp inhibition.

## Background

Positron emission tomography (PET) is a powerful molecular imaging technique that has earned an established position in the clinic as a diagnostic tool. It provides a combination of high sensitivity, tissue penetration and quantitativity that is unmatched by any other molecular imaging technique [1]. Apart from its clinical applications, PET is also frequently applied in translational research and drug development to non-invasively quantify biological targets, dose–occupancy relationships or therapeutic response [2, 3].

During preclinical development of novel PET tracers, it is important to use animals that represent human physiology as closely as possible. Rodents are usually tested first, for a number of reasons including lower costs compared to larger animals and the availability of disease models [4]. Compared to other species, rodents have been demonstrated to have increased brain expression of the efflux transporter, P-glycoprotein (P-gp), both in absolute terms and relative to other drug efflux transporters [5, 6]. P-gp is an ATP-dependent efflux pump, localized at the luminal side of the brain capillary endothelium which forms the blood–brain barrier (BBB). Substrates of P-gp are pumped out into the lumen of the brain capillaries and thus removed from the brain tissue [7]. P-gp activity is known to be a major factor limiting the brain uptake of PET tracers in rodents [8, 9]. If tracers for central nervous system (CNS) targets are initially evaluated in rodents and show low brain uptake, they could be de-selected for further translation, even though they would have worked in higher species, including humans.

We have previously demonstrated that the brain uptake of two PET tracers used for brain receptor imaging, [^11^C]GR205171 and [^18^F]Altanserin, was three–sixfold lower in rats than in humans, which is unsatisfactorily low [10]. In the same study, the uptake of [^18^F]Altanserin in the minipig brain was 3.8-fold higher than in the rat brain, apparently due to a lower P-gp activity in pigs. Detecting brain uptake of this magnitude during initial evaluation of a tracer would warrant further translation to humans.

In this study, we investigate if rodents generally display higher efflux transporter activity than pigs by studying the brain uptake of eight structurally different PET tracers in both rodents and pigs. Furthermore, we propose an experimental design which accounts for elevated P-gp activity in rodents and facilitates the translation of tracers from rodents to higher species. Specifically, we supplement the traditional evaluation of tracer uptake at baseline and after selective target block with concomitant inhibition of P-gp activity.

**Fig. 1:**
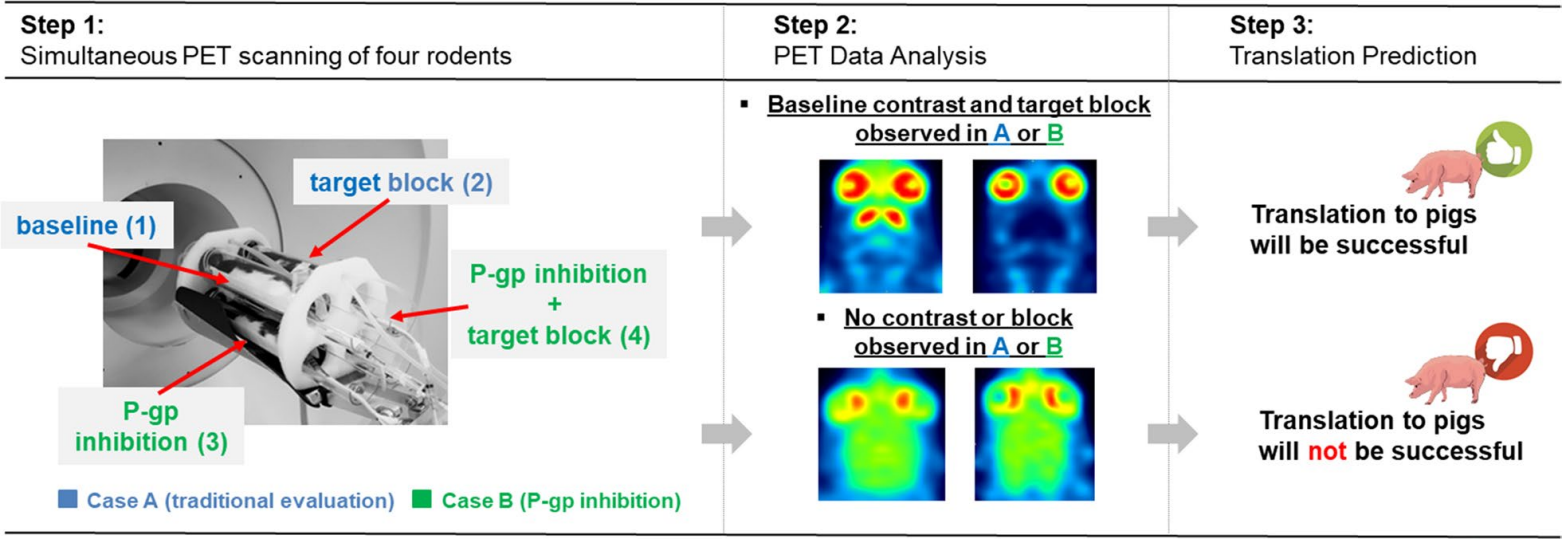
Tracer evaluation strategy. *Step 1 and 2*: Traditionally, specific binding can be determined by comparing tracer uptake at baseline and after target block (*Case A*). This work proposes to perform the same two experiments with simultaneous inhibition of P-gp (*Case B*). *Step 3*: If specific binding is observed in Case A or B, a successful translation from rodent to pig is predicted

Figure 1 summarizes our set-up. In short, rats were scanned under four experimental conditions: drug-naïve or “baseline” (1), target block (2), P-gp inhibition (3) and target block combined with P-gp inhibition (4). The advantage of this set-up is that, in addition to assessing the specific binding of the tracer, it determines whether the tracer is a P-gp substrate or not. Importantly, this set-up is compatible with the higher-throughput scanning approach earlier reported by us [11], where four rats are scanned simultaneously in a clinical PET scanner. Thus, all four experimental conditions can be evaluated in a single scan.

Eight PET tracers for three different receptor systems were used in this study. Serotonin 5-HT_2A_ receptor tracers [^18^F]Altanserin, [^11^C]Cimbi-36, [^11^C]Pimavanserin and [^18^F]MH.MZ, serotonin 5-HT_7_ receptor tracers [^11^C]Cimbi-701, [^11^C]Cimbi-717 and [^11^C]BA-10 and dopamine D_2/3_ receptor tracer [^18^F]Fallypride were studied.

Results of the tracer evaluation via both the traditional workflow (baseline and target block conditions) and the new workflow (P-gp inhibition alone and combined with target block) were used to predict the performance of the same tracer in pigs. The prediction algorithm is illustrated in Additional file 1: Fig. S1. We hypothesized that additional evaluation of a tracer’s P-gp dependency would give a better prediction of its performance in pigs and ultimately in humans than the traditional workflow.

## Methods

### Radiochemistry

[^18^F]Altanserin, [^11^C]Cimbi-36, [^11^C]Pimavanserin, [^18^F]MH.MZ, [^18^F]Fallypride, [^11^C]Cimbi-701, [^11^C]Cimbi-717 and [^11^C]BA-10 were synthesized as previously reported [12–19]. Chemical structures of all compounds are displayed in the Additional file (Additional file 1: Fig. S2).

### Animals

Long-Evans WT (or Sprague–Dawley in the case of [^18^F]Altanserin) female rats of 200–300 g (8–10 weeks) (Charles River, Calco, Italy) were housed in groups of 2–3 animals per cage in a climate-controlled rodent facility with a 12-h light/dark cycle. The animals had free access to water and were fed ad libitum. All procedures were conducted in accordance with the European Commission’s Directive 2010/63/EU, FELASA and ARRIVE guidelines for animal research and, with approval from The Danish Council for Animal Ethics (license numbers: 2017-15-0201-01283, 2012-15-2934-00156, 2007-561-1320) as well as the Department of Experimental Medicine, University of Copenhagen.

### Pig PET experiments

Description of PET experiments in pigs and analysis of obtained data can be found in the Additional file 1.

### Rat PET scanning protocol

On the day of scanning, rats were transported to the scanner at least one hour prior to the experiment. Isoflurane, 3–3.5% in 0.6% oxygen, was used to induce anaesthesia, while anaesthesia was maintained with 2.0–2.5% isoflurane during the scans. The PET tracers were administered as intravenous (i.v.) bolus injections via tail vein catheters (BD Neoflon 25G, Stockholm, Sweden) at the beginning of the scan with doses being between 5–20 MBq. The rats were subsequently scanned in the High-Resolution Research Tomograph (HRRT) scanner (Siemens AG, Munich, Germany) using a custom-made 2 × 2 rat holder, which enabled simultaneous scanning of four rats (Fig. 1) [11]. The animals were scanned for either 60 min (^11^C) or 90 min (^18^F), followed by a transmission scan at speed 10 (acquisition time approximately 6 min). The animals were scanned at baseline (no pre-treatment before tracer injection) and after receiving P-gp inhibition with or without target block. Details of inhibition and blocking regimens are described in Table 1. For combined inhibition and target block scans, rats were pre-treated with the P-gp inhibitor elacridar (5 mg/kg, Carbosynth, Compton, UK) and the 5-HT_7_ receptor antagonist SB-269970 (3 mg/kg, Tocris Bioscience, Abingdon, UK), the sigma and dopamine D_2/3_ receptor antagonist haloperidol (1 mg/kg, Janssen-Cilag, Birkerød, Denmark) or the 5-HT_2A_ receptor antagonist ketanserin (3 mg/kg, Sigma-Aldrich, Saint Louis, Missouri, USA). Elacridar was given 30 min prior to tracer injection through the intravenous catheter, and receptor blocking drugs were given 15 min before tracer injection. Chosen dosages and pre-treatment intervals were based on literature data [20–23].

**Table 1:** Target blocking drugs, P-gp inhibitors and PET image summation timespan for rat PET experiments

Tracer	Target blocking drug	Dose (mg/kg)	P-gp inhibitor	Dose	Time span for image summation (min)
[^18^F]MH.MZ	Ketanserin	3	Elacridar	5	5–90
[^18^F]Altanserin	Ketanserin	3	Cyclosporin A	22.5^a^	5–90
[^11^C]Pimavanserin	Ketanserin	3	Elacridar	5	5–60
[^11^C]Cimbi-36	Ketanserin	3	Elacridar	5	5–60
[^11^C]Cimbi-717	SB269970	3	Elacridar	5	2–60
[^11^C]Cimbi-701	SB269970	3	Elacridar	5	2–60
[^11^C]BA-10	SB269970	3	Elacridar	5	30–60
[^18^F]Fallypride	Haloperidol	1	Elacridar	5	5–90

^a^After bolus injection of 22.5 mg/kg cyclosporin A, rats received constant infusion of 7.5 mg/kg/h

Studies with [^18^F]Altanserin were performed in a MicroPET Focus 120 scanner (Siemens Medical Solutions, Malvern, PA, USA). Hyponorm/midazolam (VetaPharma Ltd., Leeds, UK/Hameln Pharmaceuticals, Hameln, Germany) was used as anaesthesia. The rats received 11 ± 2 MBq of the tracer as a bolus injection. Three of the rats were administered with 22.5 mg/kg bolus of cyclosporin A (Sandimmun, Novartis, Basel, Switzerland) followed by a constant infusion of 7.5 mg/kg/h, starting 20–25 min before radiotracer administration, as previously described [10].

### Rat PET image reconstruction

For HRRT scans (all tracers except [^18^F]Altanserin), 60-min list-mode PET data were transformed into 33 dynamic frames (6 × 10, 6 × 20, 6 × 60, 8 × 120, and 7 × 300 s), while 90-min list-mode PET data were transformed into 35 dynamic frames (6 × 10, 8 × 30, 5 × 60, and 16 × 300 s). Attenuation maps were reconstructed from transmission scans using maximum a posteriori algorithm for transmission data (MAP-TR) with human head (HH) segmentation/thresholding scans [11]. All images were reconstructed using ordinary Poisson 3D ordered subset expectation maximization (OP-OSEM3D) algorithm with point spread function modelling. PET image frames consisted of 207 planes of 256 × 256 voxels of 1.22 × 1.22 × 1.22 mm. [^18^F]Altanserin scans were performed on the Focus 120 camera. Ninety-minute list-mode PET data were transformed into 25 dynamic frames (10 × 30, 5 × 120, 5 × 30, and 5 × 600 s) and reconstructed using the filtered back-projection method. Reconstructed PET image frames consisted of 95 planes of 128 × 128 voxels of 0.87 × 0.87 × 0.80 mm.

### Quantification of rat PET data

For data analysis, the software PMOD 3.7 (PMOD Technologies, Zürich, Switzerland) was used. Summed PET images were generated based on all counts recorded in the time intervals specified in Table 1. Images were then aligned to a standardized MRI-based atlas of the rat brain [24] from where pre-defined regions of interest (ROIs) were extracted. Regions known to possess high densities of relevant receptors were selected as target ROIs: medial prefrontal cortex (mPFC) and frontal cortex (FC) for 5-HT_2A_ receptor PET tracers [25], thalamus (Tha) for 5-HT_7_ receptor PET tracers [16] and striatum (Str) for dopamine D_2/3_ receptor tracer [26]. Cerebellum region (Cb), having low densities of receptors targeted by all investigated tracers, was chosen as a reference region (Additional file 1: Fig. S3). In addition, whole brain (Wb) ROI was chosen to monitor overall tracer penetration into the brain. The time–activity curves (TACs) for target ROIs were extracted from the PET images, and the activity was converted into standardized uptake values (SUV). SUV, expressed in g/mL, is equal to the concentration of radioactivity measured in the ROI divided by the injected radioactivity dose per body weight. Area under the curve (AUC) values were calculated from the TACs using GraphPad Prism 7 (GraphPad Software, California, USA) and expressed in min × g/mL. As scanning durations were different for ^11^C-labelled and ^18^F-labelled tracers (60 min and 90 min, respectively), AUC values of ^18^F-labelled tracers were calculated for both the full duration and the first 60 min of the scan.

### Rat data analysis

Apparent target-specific binding of the tracers in the target regions was expressed as specific binding ratios (SBR), which were calculated from mean full scan length AUC values applying Eq. 1; cerebellum was used as a reference region for all tracers. Changes in apparent specific binding of the tracers in response to target receptor blockade were calculated from SBR values using Eq. 2; SBR changes were calculated for “baseline—target block” and “P-gp inhibition alone—combined P-gp inhibition and target block” condition pairs. Changes in tracer uptake in the target-rich region in response to P-gp inhibition were calculated from mean AUC values for the first 60 min of the scan as shown in Eq. 3.

1 $${\text{SBR}} = \frac{{{\text{AUC}}_{{{\text{targetROI}}}} - {\text{AUC}}_{{{\text{referenceROI}}}} }}{{{\text{AUC}}_{{{\text{referenceROI}}}} }}$$

2 $$\% {\text{SBRchange}} = 100\% \times \frac{{{\text{SBR}}_{{{\text{baseline}}}} - {\text{SBR}}_{{{\text{block}}}} }}{{{\text{SBR}}_{{{\text{baseline}}}} }}$$

3 $$\% {\text{AUCchange}} = 100\% \times \frac{{{\text{AUC}}_{{\text{Pgp inhibition}}}^{60\min } {-}{\text{AUC}}_{{{\text{baseline}}}}^{60\min } }}{{{\text{AUC}}_{{{\text{baseline}}}}^{60\min } }}$$

Evaluation of a tracer was considered successful if (whether with or without P-gp inhibition) the tracer showed an SBR value of at least 0.15 (15% higher uptake in the target region relative to reference region), and this SBR value decreased by at least 30% under target block condition.

## Results

**Fig. 2:**
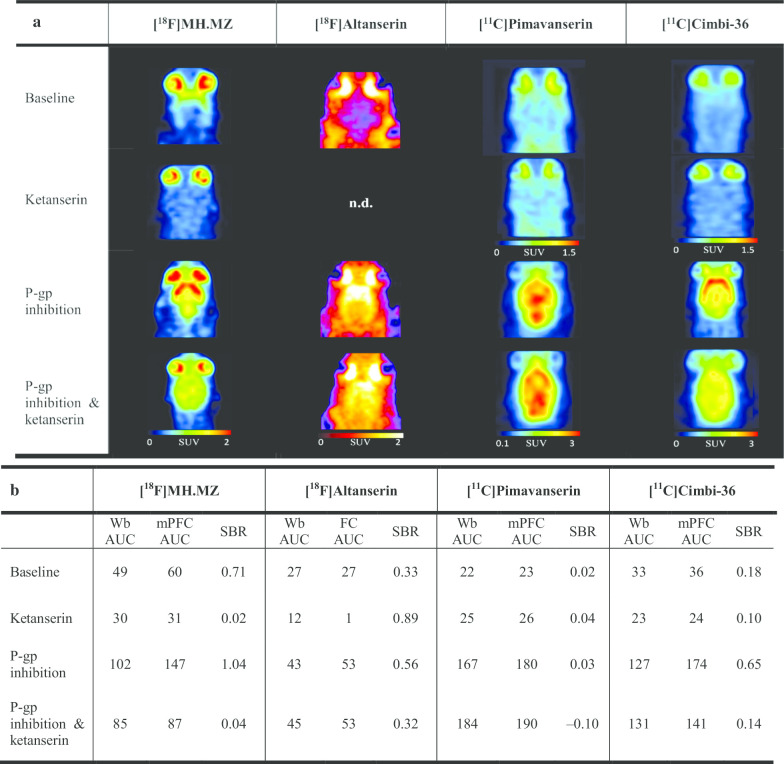
PET images (**a**), AUC and SBR values (**b**) for the 5-HT_2A_ receptor tracers [^18^F]MH.MZ, [^18^F]Altanserin, [^11^C]Pimavanserin and [^11^C]Cimbi-36. Representative horizontal rat PET images, 60-min AUC values (min × g/mL) and SBR values are shown for baseline, target block with ketanserin, efflux transporter inhibition and combined P-gp inhibition—target block conditions (see Table 1 for details on drug dosing and PET image summation). n.d. = summed PET image could not be generated. Numbers of rats scanned per experimental condition are shown in Additional file 1: Table S2. *Wb* whole brain, *mPFC* medial prefrontal cortex, *FC* frontal cortex, *AUC* area under the curve, *SBR* specific binding ratios, *P-gp* P-glycoprotein

Of the four 5-HT_2A_ receptor tracers investigated, only [^18^F]MH.MZ showed substantial uptake in the rat brain at baseline: AUC for the target receptor-rich mPFC was 60 ± 5 min × g/mL (here and further AUC values refer to the first 60 min of the scan unless stated otherwise; full scan length AUC values for all tracers, ROIs and experimental conditions are presented in Additional file 1: Table S1; numbers of rats scanned per experimental condition are shown in Additional file 1: Table S2). Baseline SBR for mPFC equalled 0.71. Pre-treatment with ketanserin, a 5-HT_2A_ receptor antagonist, decreased SBR to 0.02 (− 97% change). Under P-gp inhibition with elacridar, the AUC for the mPFC rose to 147 min × g/mL (+ 143%), and the SBR value for the mPFC reached 1.04. Combining target blockade with P-gp inhibition decreased the SBR to 0.04 (− 96%). Thus, the 5-HT_2A_ receptor-specific binding and the blocking of it were clearly detectable both with and without P-gp inhibition for [^18^F]MH.MZ (Fig. 2 and Additional file 1: Fig. S4).

In contrast, [^18^F]Altanserin, [^11^C]Pimavanserin and [^11^C]Cimbi-36 showed low brain penetration at baseline, with AUC values for mPFC (FC for [^18^F]Altanserin) in the range of 23–36 min × g/mL (Fig. 2 and Additional file 1: Fig. S4). SBR values were 0.33 for [^18^F]Altanserin, 0.02 for [^11^C]Pimavanserin and 0.18 for [^11^C]Cimbi-36. After ketanserin pre-treatment, [^11^C]Cimbi-36 SBR fell to 0.10 (–44%), while SBR values for [^18^F]Altanserin and [^11^C]Pimavanserin actually grew to 0.89 (+ 172%) and 0.04 (+ 110%), respectively. Thus, for these three tracers, blockable target-specific binding could only be detected for [^11^C]Cimbi-36.

After P-gp inhibition, mPFC AUC values for [^18^F]Altanserin, [^11^C]Pimavanserin and [^11^C]Cimbi-36 increased to 53 min × g/mL (+ 93%), 180 min × g/mL (+ 689%) and 174 min × g/mL (+ 391%), while SBR values reached 0.56, 0.03 and 0.65, respectively. Ketanserin blockade combined with P-gp inhibition resulted in SBR values of 0.32 (− 42%) for [^18^F]Altanserin and 0.14 (− 79%) for [^11^C]Cimbi-36. For [^11^C]Pimavanserin, AUC in the cerebellum after combined ketanserin and elacridar pre-treatment was greater than AUC in the mPFC, resulting in a negative SBR value of –0.10. However, a change in SBR from 0.03 to –0.10, although high in relative terms (-395%), can hardly be interpreted as a sign of target-specific binding because both values are very low.

All in all, for [^18^F]Altanserin, 5-HT_2A_ receptor-specific binding and the blocking effect became observable after P-gp inhibition, for [^11^C]Cimbi-36 the use of P-gp inhibition led to an amplification of both apparent specific binding at baseline and the blocking effect, while [^11^C]Pimavanserin did not show any sign of specific 5-HT_2A_ receptor binding either with or without P-gp inhibition (Fig. 2a).

**Fig. 3:**
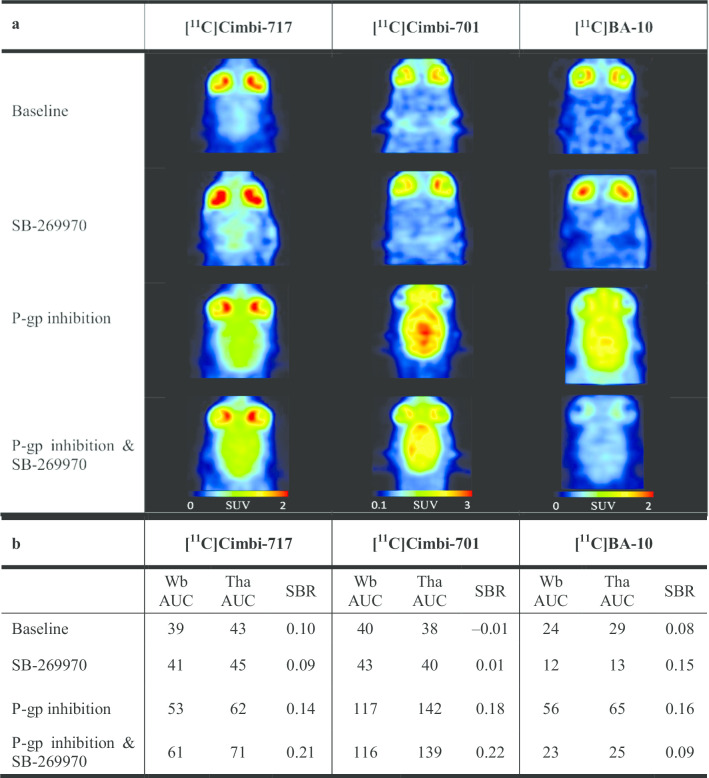
PET images (**a**), AUC and SBR values (**b**) for the 5-HT_7_ receptor tracers [^11^C]Cimbi-717, [^11^C]Cimbi-701, and [^11^C]BA-10. Representative horizontal rat PET images, 60-min AUC values (min × g/mL) and SBR values are shown for baseline, target block with SB-269970, P-gp inhibition with elacridar and combined P-gp inhibition—target block conditions (see Table 1 for details on drug dosing and PET image summation). Numbers of rats scanned per experimental condition are shown in Additional file 1: Table S2. *Wb* whole brain, *Tha* thalamus, *Cb* cerebellum, *AUC* area under the curve, *SBR* specific binding ratios, *P-gp* P-glycoprotein

Among tracers targeting 5-HT_7_ receptors, we investigated [^11^C]Cimbi-717, [^11^C]Cimbi-701 and [^11^C]BA-10 [16, 17, 27]. At baseline, all tracers showed low AUC values between 30 and 43 min × g/mL in the 5-HT_7_ receptor-rich thalamus (Fig. 3 and Additional file 1: Fig. S5). Baseline thalamic SBR values were also low for all three tracers, varying from –0.01 for [^11^C]Cimbi-701 to 0.10 for [^11^C]Cimbi-701. After pre-treatment with the 5-HT_7_ selective antagonist SB-269970, the SBR values for [^11^C]Cimbi-701 and [^11^C]BA-10 increased, while for [^11^C]Cimbi-717 the SBR value decreased to 0.09 (at − 5%). Thalamic AUC values reached 62 min × g/mL (+ 47%) for [^11^C]Cimbi-717, 142 min × g/mL (+ 277%) for [^11^C]Cimbi-701 and 65 min × g/mL (+ 121%) for [^11^C]BA-10 after P-gp inhibition with elacridar. Respective SBR values were 0.14, 0.18 and 0.16. Simultaneous inhibition of P-gp and blocking of the 5-HT_7_ receptor decreased [^11^C]BA-10 SBR to 0.09 (–44%), while SBRs for [^11^C]Cimbi-717 and [^11^C]Cimbi-701 actually increased to 0.21 (+ 46%) and 0.22 (+ 23%), respectively. In summary, specific binding could only be detected for [^11^C]BA-10 after P-gp inhibition.

**Fig. 4:**
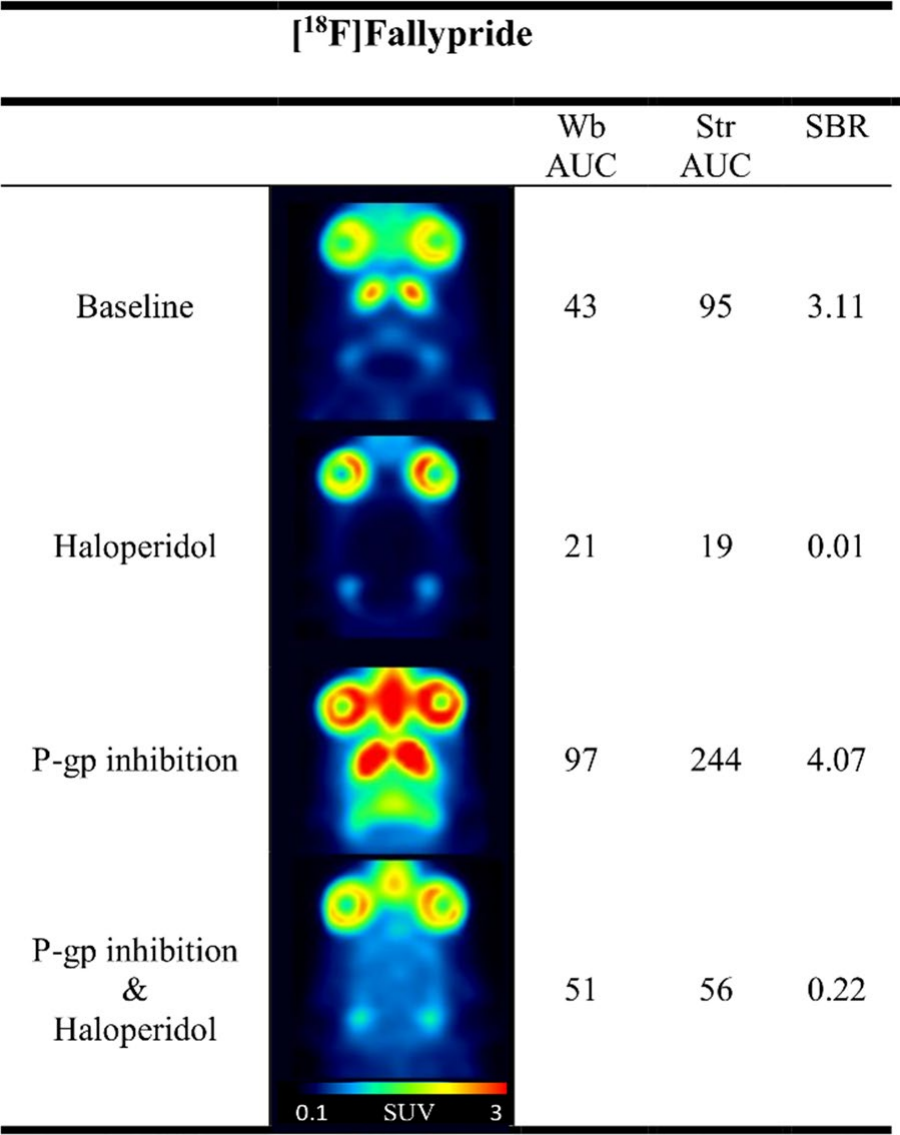
PET images, AUC and SBR values for the D_2/3_ receptor tracer [^18^F]Fallypride. Representative horizontal rat PET images, 60-min AUC values (min × g/mL) and SBR values are shown for baseline, target block with haloperidol, P-gp inhibition with elacridar and combined P-gp inhibition—target block conditions (see Table 1 for details on drug dosing and PET image summation, *n* = 2). *Str* striatum, *Cb* cerebellum, *Wb* whole brain, *AUC* area under the curve, *SBR* specific binding ratios, *P-gp* P-glycoprotein

The dopamine D_2/3_ receptor ligand [^18^F]Fallypride was included into the study as a non-serotonergic tracer. Baseline striatal AUC equalled 95 min × g/mL. Striatal SBR was 3.11 at baseline and fell to 0.01 (− 100%) after haloperidol pre-treatment (Fig. 4 and Additional file 1: Fig. S5). After P-gp inhibition, striatal AUC rose to 244 min × g/mL (+ 158%), and SBR reached 4.07. Combined P-gp inhibition and target (D_2/3_) block decreased striatal SBR to 0.22 (− 95%). Therefore, target-specific binding was clearly visible both without and with P-gp inhibition.

Evaluation of [^18^F]MH.MZ, [^18^F]Altanserin, [^11^C]Pimavanserin, [^11^C]Cimbi-36, [^11^C]Cimbi-717 and [^11^C]Cimbi-701 in pigs was reported previously [15, 19, 27, 28]. Time–activity curves for [^11^C]BA-10 and [^18^F]Fallypride in pig brain are presented in Additional file 1: Figs. S6 and S7, respectively.

## Discussion

In this work, we propose a higher-throughput approach to improve the translation of CNS PET tracers from rodents to higher species. Our data suggest that one of the main factors limiting translation of CNS tracers from rats to larger animals is the higher activity of efflux pumps, including P-gp, in rats. We used cyclosporine A and elacridar to inhibit the action of P-gp, the dominant drug efflux transporter in the rodent brain and study the influence of P-gp on the accumulation of a set of eight tracers [5]. Cyclosporine A is a selective P-gp inhibitor, while elacridar inhibits both P-gp and BCRP (breast cancer resistance protein) efflux pumps [29]. Within our data set, all investigated tracers showed higher brain uptake in rats when the action of P-gp was inhibited. The smallest increase in target-rich region AUC values after P-gp inhibition was observed for [^11^C]Cimbi-717 (47%) and [^18^F]Altanserin (93%), while for all other tracers, AUC values increased by 100–700%. Brain uptake of [^18^F]Altanserin was previously reported to increase 2.6-fold (by 160%) in response to P-gp inhibition [10], which is higher but still comparable to the results obtained in this work (+ 93%), using the same P-gp inhibition condition. Higher increase reported in [10] could have been the consequence of a higher body weight of the rats used for the experiments (370 g vs 200–300 g in this work). It should be noted that all compounds evaluated by us are secondary and/or tertiary amines, which tend to be P-gp substrates [30, 31]. However, an amino group is often present in CNS PET tracers.

In the absence of P-gp inhibition, only [^18^F]MH.MZ, [^11^C]Cimbi-36 and [^18^F]Fallypride showed preferential accumulation (SBR values of > 0.15) in target-rich regions and more than 30% reduction in SBR values after a selective target block (Figs. 2 and 4). [^18^F]Altanserin demonstrated a decent SBR value (0.33) at baseline without P-gp inhibition, but ketanserin pre-treatment led to a drastic decrease of tracer uptake in both frontal cortex and cerebellum, with uptake in the cerebellum falling even more drastically (Additional file 1: Table S1). As a consequence, [^18^F]Altanserin´s SBR after target blockade was higher compared to baseline conditions. Likewise, brain uptake in the reference ROI decreased more than in the target ROI in response to target blockade for [^11^C]BA-10. These paradoxical observations may be explained by the hindered diffusion of the tracers across the BBB or their accelerated washout from the brain caused by the vasodilatory [20] or vasoconstrictive [32] properties of the respective blocking drugs (ketanserin and SB-269970). Saturation of P-gp or other efflux transporters by the blocking drugs is an unlikely explanation, because this would have increased the uptake of the tracers in both target and reference ROIs. All in all, further research is required to unequivocally resolve this issue.

We hypothesized that the target binding of tracers that are actively transported out of the rat brain can nevertheless be assessed once the increased activity of efflux pumps is inhibited. Therefore, all eight tracers were tested under P-gp inhibition with and without a simultaneous target block. P-gp inhibition led to an increase in baseline SBR values for all tracers (Additional file 1: Fig. S8). Five tracers ([^18^F]MH.MZ, [^18^F]Altanserin, [^11^C]Cimbi-36, [^11^C]BA-10, [^18^F]Fallypride) showed a pronounced reduction of apparent specific binding in target-rich regions (> 30% SBR decrease) in response to target block under these conditions. These included [^18^F]Altanserin and [^11^C]BA-10, for which no such reduction could be demonstrated using the traditional workflow. For [^11^C]Pimavanserin, [^11^C]Cimbi-717 and [^11^C]Cimbi-701, no receptor-specific binding could be detected under P-gp inhibition: target region SBR values were either too low at baseline ([^11^C]Pimavanserin, Fig. 2) or failed to decrease after target receptor blockade ([^11^C]Cimbi-717 and [^11^C]Cimbi-701, Fig. 3). For the latter two tracers, their uptake in the target and reference ROIs under P-gp inhibition either did not change in response to target receptor blockade or slightly increased in both ROIs, which led to an increase in SBR (Fig. 3). This could have been caused by the perturbations in the cerebral blood flow due to the pharmacological action of the blocking drug (SB-269970, see above) or by the influx into the brain of the excess of the tracers displaced from 5-HT_7_ receptors in peripheral tissues by the blocking drug.

Based on our data in rats, we predicted whether a tracer would work in pigs or not (see Additional file 1: Fig. S1) and compared these predictions to actual results obtained in pigs, as well as to the evaluation results in humans for those tracers that reached the clinical evaluation stage ([^18^F]MH.MZ, [^18^F]Altanserin, [^11^C]Cimbi-36 and [^18^F]Fallypride). Table 2 and Additional file 1: Table S3 summarize the outcomes. In contrast to rats (Figs. 2 and 3), low brain uptake was not an issue for any of the investigated tracers during evaluation in pigs and humans (Additional file 1: Table S3 and [33–36]). This confirms the notion that rats generally have a highly efficient brain efflux transporter system, which can limit the uptake of tracers in the brain, whereas in larger/higher species the efflux transporter system has less influence on the tracer uptake. Seven out of eight tracers displayed target-specific binding in pigs, i.e. pre-treatment with a specific receptor blocking agent reduced the binding potential (BP_ND_) of the tracer in the target region (by 30% or more). For all tracers except [^11^C]Cimbi-701 and [^11^C]Cimbi-717, which had shown specific binding in pigs but not in rats, baseline BP_ND_ values in pigs highly correlated with baseline SBR values in rats, both with and without P-gp inhibition (Additional file 1: Fig. S9). [^11^C]Pimavanserin did not show any specific binding in pigs.

**Table 2:** Short summary of tracer evaluation in rats, pigs and humans

Tracer	Target-specific binding confirmed			Prediction outcome^c^	Translated to humans
	Rat		Pig		
	Traditional^a^	P-gpI^b^			
[^18^F]MH.MZ	Yes	Yes	Yes		Yes
[^18^F]Altanserin	No	Yes	Yes		Yes
[^11^C]Pimavanserin	No	No	No		
[^11^C]Cimbi-36	Yes	Yes	Yes		Yes
[^11^C]Cimbi-717	No	No	Yes		
[^11^C]Cimbi-701	No	No	Yes		
[^11^C]BA-10	No	Yes	Yes		
[^18^F]Fallypride	Yes	Yes	Yes		Yes

Concrete estimates of target-specific binding at baseline and its changes in response to target receptor blockade in rats and pigs, as well as estimates of target-specific binding in humans are summarized in Additional file 1: Table S3

^a^Traditional evaluation workflow without P-gp inhibition

^b^P-gpI = P-gp inhibition

^c^Prediction outcome indicates if the evaluation outcome in pigs could (thumb up) or could not (thumb down) be correctly predicted from evaluation outcome in rats

In our study, blocking experiments in rats without P-gp inhibition detected specific binding for three tracers ([^18^F]MH.MZ, [^11^C]Cimbi-36 and [^18^F]Fallypride) and without additional experiments in non-rodent species, the remaining tracers would likely have been discarded. The use of P-gp inhibition helped to additionally identify two tracers ([^18^F]Altanserin and [^11^C]BA-10) that showed specific binding. [^11^C]Pimavanserin did not show any specific binding either with or without P-gp inhibition in rats, and behaved in the same way in pigs. Agreement between pig and rat data on [^11^C]Pimavanserin is a positive finding within the framework of this study, even though it may be perceived as disappointing that a ligand with demonstrated sub-nanomolar affinity and high selectivity towards 5-HT_2A_ receptors [37] turned out to be unsuitable for receptor imaging. Rodent data for [^11^C]Cimbi-701 and [^11^C]Cimbi-717, on the other hand, demonstrate the limitations of brain radioligand evaluation in rodents: even with the use of P-gp inhibition, these tracers would have been discarded based on rodent data only. Apart from efflux transporter expression and activity, inter-species differences in receptor abundances or metabolic pathways are factors that may have a crucial impact on the translational validation of PET tracers [38, 39]. Densities of 5-HT_2A_, 5-HT_7_ and D_2/3_ receptors in the respective receptor-rich regions of rodent, pig and human brain are summarized from literature data in Additional file 1: Table S4 [28, 40–42]. Densities of 5-HT_7_ receptors in rat and pig thalamus are very close, so the differences in metabolism between rats and pigs are a more likely explanation for the discrepant results of [^11^C]Cimbi-701 and [^11^C]Cimbi-717 evaluation in these two species.

## Conclusions

The inclusion of P-gp inhibition into the workflow helped to predict the outcome of six out of eight cases (75% success rate), even though all tracers were strong substrates of P-gp. A traditional evaluation workflow without P-gp inhibition could only predict the outcome for four out of eight tracers (50% success rate). Our results demonstrate how addition of P-gp inhibition can aid in the interpretation of initial tracer evaluation in rats, improve the translatability and minimize unjustified discontinuations of promising tracers. We believe that the proposed workflow allows for a more effective initial in vivo screening using rats.

## Supplementary information

**Additional file 1**. Detailed overview of the experimental design, description of experiments in pigs, chemical structures of all studied compounds, investigated brain regions on the MRI template, time–activity curves for rat and pig ([^18^F]Fallypride and [^11^C]BA-10) scans, SBR and AUC values for all tracers, numbers of rats scanned per experimental condition are detailed in the Additional file.

## Data Availability

The data sets generated during and/or analysed during the current study are available from the corresponding author on reasonable request.

## Abbreviations

5-HT_2A_: Serotonin receptor of subtype 2A
5-HT_7_: Serotonin receptor of subtype 7
ARRIVE: Animal Research: Reporting of In Vivo Experiments
ATP: Adenosine triphosphate
AUC: Area under the curve
BBB: Blood–brain barrier
BPND: Binding potential relative to non-displaceable uptake
Cb: Cerebellum
CNS: Central nervous system
D_2/3_: Dopamine receptors of subtypes 2 and 3
FC: Frontal cortex
FELASA: Federation for Laboratory Animal Science Associations
HH: Human head
HRRT: High-Resolution Research Tomograph
MAP-TR: Maximum a posteriori algorithm for transmission data
mPFC: Medial prefrontal cortex
MRI: Magnetic resonance imaging
OP-OSEM3D: Ordinary Poisson 3D ordered subset expectation maximization
PET: Positron emission tomography
P-gp: P-glycoprotein
ROI: Region of interest
SBR: Specific binding ratio
Str: Striatum
TAC: Time–activity curve
Tha: Thalamus
Wb: Whole brain
WT: Wild-type
