# Supplementary material for: Blocking of efflux transporters in rats improves translational validation of brain radioligands
Source: EJNMMI Res. 2020 Oct 19;10:124. doi: 10.1186/s13550-020-00718-x (PMC7572968; doi:10.1186/s13550-020-00718-x)
Supplement: Supplementary file 1 — Additional file 1. Detailed overview of the experimental design, description of experiments in pigs, chemical structures of all studied compounds, investigated brain regions on the MRI template, time–activity curves for rat and pig ([18F]Fallypride and [11C]BA-10) scans, SBR and AUC values for all tracers, numbers of rats scanned per experimental condition are detailed in the Additional file. [file 13550_2020_718_MOESM1_ESM.docx]

**Blocking of Efflux Transporters in Rats Improves Translational Validation of Brain Radioligands**

Vladimir Shalgunov^1,2,#^, Mengfei Xiong^1,3,4,#^, Elina T. L’Estrade^1,3,5^, Nakul R. Raval^3^, Ida V. Andersen^1,3^, Fraser G. Edgar^1^, Nikolaj R. Speth^3^_,_ Simone L. Baerentzen^3^, Hanne D. Hansen^3,6^, Lene L. Donovan^3^, Arafat Nasser^3^, Siv T. Peitersen^3^, Andreas Kjaer^2,7^, Gitte M. Knudsen^3^, Stina Syvänen^4^, Mikael Palner^3,8^, Matthias M. Herth^1,2^*

1. Department of Drug Design and Pharmacology, Faculty of Health and Medical Sciences, University of Copenhagen, Jagtvej 160, 2100 Copenhagen, Denmark
2. Department of Clinical Physiology, Nuclear Medicine & PET, Copenhagen University Hospital, Rigshospitalet, Blegdamsvej 9, 2100 Copenhagen, Denmark
3. Neurobiology Research Unit and CIMBI, Rigshospitalet and University of Copenhagen, Blegdamsvej 9, 2100 Copenhagen, Denmark
4. Department of Public Health and Caring Sciences/Geriatrics, Uppsala University, Rudbeck Laboratory, 75185 Uppsala, Sweden
5. Radiation Physics, Nuclear Medicine Physics Unit, Skånes University Hospital, Barngatan 3, Lund 222 42, Sweden
6. A. A. Martinos Center for Biomedical Imaging, Massachusetts General Hospital, 149 13^th^ Street, 02129 Charlestown, MA, USA
7. Cluster for Molecular Imaging, Department of Biomedical Sciences, Faculty of Health and Medical Sciences, University of Copenhagen, Blegdamsvej 3, 2200 Copenhagen, Denmark
8. Center for Translational Neuromedicine, Faculty of Health and Medical Sciences, University of Copenhagen, Blegdamsvej 3B, 2200 Copenhagen, Denmark.

^#^ These authors contributed equally to this work.

* Corresponding author

Matthias Herth

Associate Professor, University of Copenhagen, Department of Drug Design and Pharmacology

e-mail: [matthias.herth@sund.ku.dk](mailto:matthias.herth@sund.ku.dk)

ORCID: 0000-0002-7788-513X

**Keywords**: P-gp, efflux transporter, PET, rodents, pigs, translation, species differences

**Table of Contents**

[Pig PET experiments 3](#_Toc44450529)

[Additional file 1: Figures 5](#_Toc44450530)

[Additional file 1: Tables 12](#_Toc44450531)

[References 15](#_Toc44450532)

# Pig PET experiments

Two female pigs (crossbreed of Landrace × Yorkshire × Duroc) weighing on average 20 kg (approx. 9 weeks old) were used in the present study. Animals were sourced from local farms and acclimatized for 7–9 days in an enriched environment prior to experiments. Pig anesthesia was induced approximately 3 h prior to scanning by i.m. injection of 0.13 ml/kg zoletil veterinary mixture (11.36 mg/mL xylazine, 11.36 mg/ml ketamine, 1.82 mg/ml butorphanol, 1.82 mg/ml methadone) and maintained by 15 mg/kg/h propofol infusion i.v.. Femoral arteries, ear veins and mammary veins were used for intra vascular access. Endotracheal intubation allowed for ventilation with 20% oxygen in air at 10 ml/kg. Urine catheter was placed to avoid discomfort and stress. The animals were closely monitored throughout the experiment, with peripheral O_2_ and end-tidal CO_2_ saturation, heart rate, blood pressure, blood glucose and temperature. Pigs were scanned in the HRRT scanner (Siemens AG, Munich, Germany). One animal was scanned at baseline with [^18^F]fallypride (injected activity = 518 MBq, injected mass = 5.61 µg). A second animal was scanned with [^11^C]BA-10 at baseline (injected activity = 262 MBq, injected mass = 0.51 µg) and after pretreatment (injected activity = 517 MBq injected mass = 0.58 µg) with the 5-HT_7_ receptor antagonist SB-269970 (1 mg/kg/h, Tocris Bioscience, Abingdon, United Kingdom) Infusion of SB-266970 was initiated 30 min prior to the injection of the tracer.

Manual arterial blood samples for the [^11^C]BA-10 scans were drawn at 2.5, 5, 10, 20, 30, 45, 60 and 90 min after injection, while an ABSS autosampler (Allogg Technology, Sweden) continuously measured arterial whole blood radioactivity during the first 30 min. Manual blood samples were used for the measurements of total radioactivity in whole blood and plasma using a gamma well counter (Cobra 5003; Packard Instruments, Meriden, USA), which was cross-calibrated to the HRRT scanner and to the autosampler. Radiolabeled parent compound and metabolites were measured in plasma using HPLC with column switching and online radioactivity detection as previously described [1].

**PET image reconstruction:** For [^11^C]BA-10 scans, 90-minute list-mode PET data was reconstructed into 38 dynamic frames of increasing length (6 × 10, 6 × 20, 4 × 30, 6 × 60, 2 × 180, 8 × 300, and 3 × 600 s). For [^18^F]fallypride scans, 150-minute list-mode PET data was reconstructed into 58 dynamic frames of increasing length (6 × 10, 6 × 20, 6 × 30, 9 × 60, 4 × 120, 16 × 300, 8 × 150 and 3 × 300 s). While a similar reconstruction method for the two tracers used an ordinary Poisson three-dimensional ordered-subset expectation maximization with point spread function modeling (OP-3D-OSEM-PSF), 16 subsets and 10 iterations with all standard corrections [2,3]. Attenuation correction was done using the HRRT maximum a posteriori transmission reconstruction method (MAP-TR) μ-map [4]. Images consist of 207 planes of 256 × 256 voxels of 1.22 × 1.22 × 1.22 mm in size. Brain parcellation was performed as previously described [5].

**Data analysis**: PMOD 3.0 (PMOD Technologies, Switzerland) was used for kinetic modeling. BP_ND_ values for [^18^F]Fallypride were calculated using a simplified refernce tissue model (SRTM) with the Cerebellum (non-vermis) as a reference region [6,7]. V_T_ values for the [^11^C]BA-10 were calculated using the 1 tissue compartment (1TC) model. We calculated the occupancy of SB-269970 and non-displaceable volume of distribution (V_ND_) using the V_T_ values from the 1TC model in a Lassen plot [8]. Nondisplaceable-uptake-normalized binding potentials (BP_ND_) were determined by reverse calculations from 1TC V_T_’s using Equation I:

$\text{BP}_{\text{ND}}\text{=}\frac{\text{(}\text{V}_{\text{T}}\text{-}\text{V}_{\text{ND}}\text{)}}{\text{V}_{\text{ND}}}$ (Eq. I)

# Additional file 1: Figures


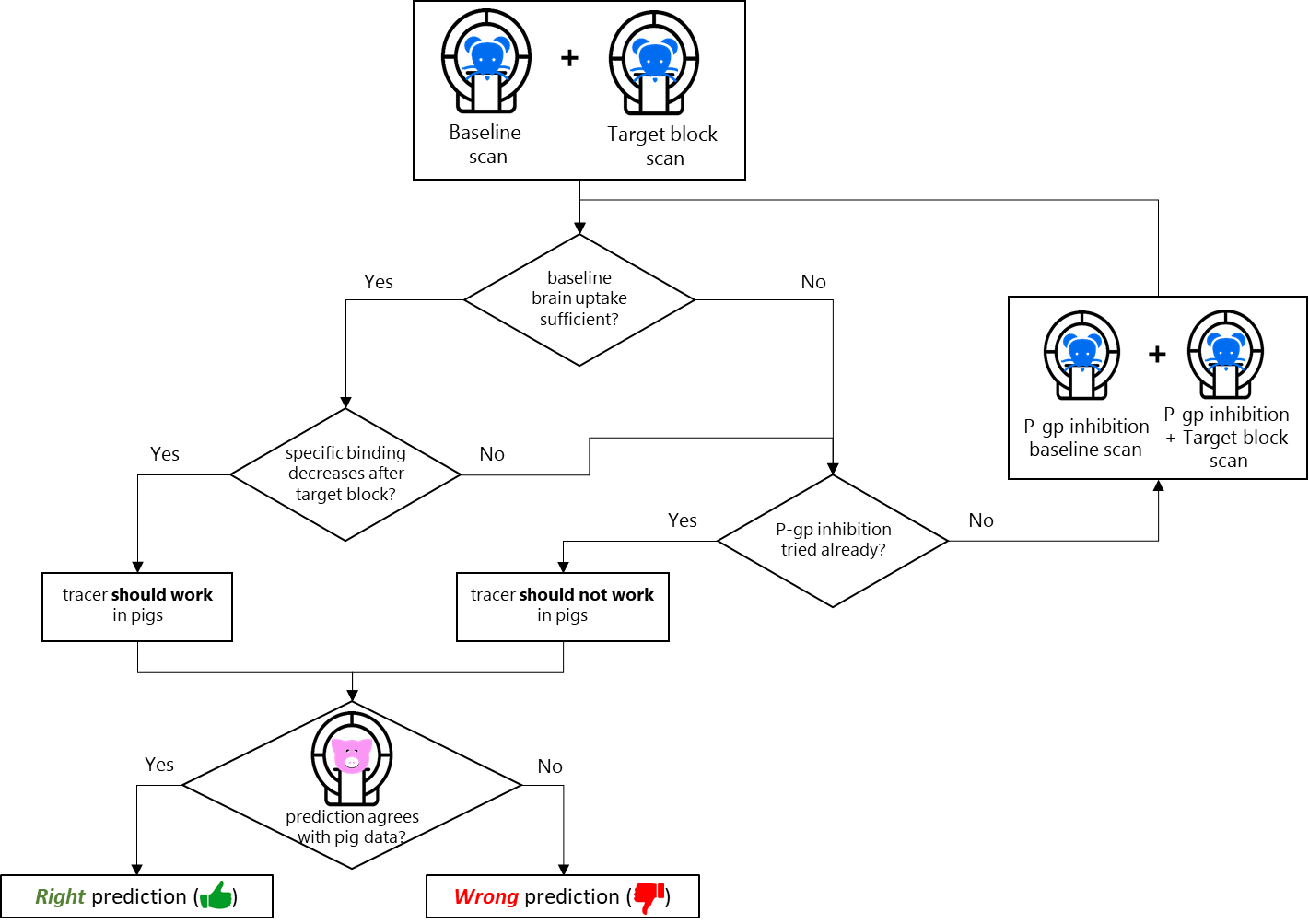


**Additional file 1: Fig. S1**. Experimental design and decision making tree.

First, tracer uptake is evaluated in rats at baseline and target block conditions without P-gp inhibition. If brain uptake at baseline is too low, or no blocking of apparent specific binding can be observed, new baseline and target block scans in rats are performed, now with inhibition of P-gp action. If, either with or without P-gp inhibition, the tracer shows preferential accumulation in the target-rich region, which decreases after target block, a prediction is made that sufficient brain uptake and specific target binding should also be observed in pigs, i.e. the tracer “should work”. If no specific binding can be detected by comparing baseline and block scans in rats either with or without P-gp inhibition, or if baseline tracer uptake stays low even after P-gp inhibition, the tracer is predicted not to work in pigs.

Icons are modified by Freepik from Flaticon.

**Additional file 1: Fig. S2**. Chemical structures of all studied compounds.

[^18^F]MH.MZ

[^18^F]Altanserin

[^11^C]Cimbi-36

[^11^C]Pimavanserin

[^11^C]Cimbi-717

[^11^C]Cimbi-701

[^18^F]Fallypride

[^11^C]BA-10

**A**


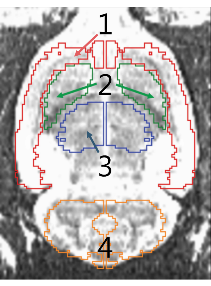


| **B** | Receptor-rich regions | Reference region | Whole brain |
| --- | --- | --- | --- |
| [^18^F]MH.MZ | mPFC | Cb | Wb |
| [^18^F]Altanserin | FC | Cb | Wb |
| [^11^C]Pimavanserin | mPFC | Cb | Wb |
| [^11^C]Cimbi-36 | mPFC | Cb | Wb |
| [^11^C]Cimbi-717 | Tha | Cb | Wb |
| [^11^C]Cimbi-701 | Tha | Cb | Wb |
| [^18^F]ENL30 | Tha | Cb | Wb |
| [^11^C]BA-10 | Tha | Cb | Wb |
| [^18^F]Fallypride | Str | Cb | Wb |

mPFC: medial prefrontal cortex; FC: frontal cortex; Cb: cerebellum; Tha: thalamus; Str: striatum; Wb: whole brain.

**Additional file 1: Fig. S3**. A: MR image of rat brain co-registered with ROIs and rat template (W.Schiffer-FDG): cortex (1), striatum (2), thalamus (3) and cerebellum (4). B: Table of selected ROIs for all radiotracers.

**Additional file 1: Fig. S4**. Time activity curves for all radiotracers at baseline and target block conditions (left), baseline and with Pgp inhibition (middle), Pgp inhibition only and Pgp inhibition + target block regime (Right). A: [^18^F]MH.MZ, B: [^18^F]Altanserin, C: [^11^C]Cimbi-36, D: [^11^C]Pimavanserin.

**Additional file 1: Fig. S5**. Time activity curves for all radiotracers at baseline and target block condition (left), baseline and with Pgp inhibition (middle), Pgp inhibition only and Pgp inhibition + target block regime (Right). A: [^11^C]Cimbi-717, B: [^11^C]Cimbi-701, C: [^11^C]BA-10, D: [^18^F]Fallypride.

**Additional file 1: Fig. S6.** Evaluation of [^11^C]BA-10 in a pig.

A: Regional time-activity curves for thalamus and cerebellum at baseline and after receptor block with SB-269970 (1 mg/kg/h).

B: Time-activity curves for arterial plasma.

C: Percentage of unmetabolized [^11^C]BA-10 in arterial plasma.

D: Lassen plot. Linear regression and goodness-of-fit (r^2^) are indicated in the plot. Occupancy determined from the plot is 35.21% and non-displaceable distribution volume V_ND_ equals 4.28. Regions used for calculation are: thalamus, insular cortex, frontal cortex, cingulate cortex, occipital cortex, temporal cortex, somatosensory cortex, hippocampus, dorsal striatum and cerebellum.

**Additional file 1: Fig. S7.** Time-activity curves of [^18^F]fallypride in a pig.

Regional time-activity curves of [^18^F]fallypride are shown for the dorsal striatum and cerebellum at baseline.

**Additional file 1: Fig. S8.** Change in baseline SBR values in rats after P-gp inhibition plotted against the baseline SBR values without P-gp inhibition.

**B**

**A**

**Additional file 1: Fig. S9.** Baseline BP_ND_ values in pigs plotted against baseline SBR values in rats without (A) and with (B) P-gp inhibition.

Pig BP_ND_ values are found in Additional file 1: Table S3.

Data for tracers targeting serotonin 5-HT_2A_, serotonin 5-HT_7_ and dopamine D_2/3_ receptors are separated into subgroups and marked by color.

# Additional file 1: Tables

**Additional file 1: Table S1:** AUC values (min×g/mL) in the ROIs of rat brain

|  | **[^18^F]MH.MZ** | | | **[^18^F]Altanserin** | | | **[^11^C]Pimavanserin** | | | **[^11^C]Cimbi-36** | | |
| --- | --- | --- | --- | --- | --- | --- | --- | --- | --- | --- | --- | --- |
|  | mPFC | Cb | Wb | Fc | Cb | Wb | mPFC | Cb | Wb | mPFC | Cb | Wb |
| Baseline | 94.5±6.6 | 55.3±1.6 | 73.1±3.8 | 41.8±1.4 | 31.5±1.5 | 39.7±0.4 | 22.8±1.0 | 22.4±0.3 | 21.9±0.4 | 35.5±2.2 | 30.1±1.9 | 32.7±1.9 |
| Ketanserin | 42.3±2.0 | 41.4±0.7 | 41.7±0.8 | 16.7±8.5 | 8.8±4.2 | 15.9±2.2 | 25.8±0.5 | 24.9±0.6 | 24.7±0.3 | 23.7±0.7 | 21.5±0.4 | 22.6±0.3 |
| P-gp inhibition | 219±4 | 107±2 | 145±3 | 85.0±1.8 | 54.6±0.8 | 65.1±0.4 | 180±3 | 174±3 | 167±2 | 174±2 | 106±1 | 127±1 |
| P-gp inhibition & ketanserin | 110 | 106 | 108 | 76.8±1.2 | 58.1±0.8 | 64.8±0.3 | 190 | 210 | 184 | 141 | 124 | 131 |

|  | **[^11^C]Cimbi-717** | | | **[^11^C]Cimbi-701** | | | **[^11^C]BA-10** | | |
| --- | --- | --- | --- | --- | --- | --- | --- | --- | --- |
|  | Tha | Cb | Wb | Tha | Cb | Wb | Tha | Cb | Wb |
| Baseline | 42.5±1.2 | 38.8±1.0 | 38.7±1.1 | 37.8±1.1 | 38.2±1.1 | 40.4±0.6 | 29.3±0.3 | 27.2±0.2 | 23.6±1.2 |
| SB-269970 | 45.1±1.2 | 41.3±0.9 | 40.5±0.9 | 39.6±0.9 | 39.4±0.6 | 43.4±0.2 | 13.1±1.2 | 11.4±1.1 | 12.1±1.2 |
| P-gp inhibition | 62.5±1.5 | 54.8±1.0 | 53.4±0.7 | 142±4 | 121±3 | 177±3 | 64.7±0.9 | 55.6±0.6 | 56.3±0.6 |
| P-gp inhibition & SB-269970 | 71.2±1.5 | 59.0±1.2 | 61.2±0.7 | 139±2 | 114±2 | 116±1 | 25.5 | 23.3 | 23 |

|  | **[^18^F]Fallypride** | | |
| --- | --- | --- | --- |
|  | Str | Cb | Wb |
| Baseline | 146±3 | 35.4±0.6 | 60.7±0.8 |
| haloperidol | 23.9±0.5 | 23.6±0.8 | 28.1±0.7 |
| P-gp inhibition | 374±13 | 73.8±1.5 | 135±4 |
| P-gp inhibition & haloperidol | 69.2±0.8 | 56.8±0.6 | 64.2±0.6 |

AUC values calculated for the whole duration of the PET scans: 60 min for ^11^C-labeled tracers, 90 min for ^18^F-labeled tracers.

Regions of interest: mPFC – medial prefrontal cortex, FC – frontal cortex, Tha – thalamus, Str – striatum, Cb – cerebellum, Wb – whole brain.

All values presented as mean±SE. Absence of SE means the AUC value was derived from a single animal. Numbers of animals per condition are listed in Additional file 1: Table S2.

**Additional file 1: Table S2:** Number of rats scanned per experimental condition

|  | Baseline | Block | P-gp inhibition | P-gp inhibition & block |
| --- | --- | --- | --- | --- |
| [^18^F]MH.MZ | 4 | 2 | 2 | 1 |
| [^18^F]Altanserin | 3 | 3 | 3 | 3 |
| [^11^C]Pimavanserin | 2 | 2 | 2 | 1 |
| [^11^C]Cimbi-36 | 3 | 2 | 2 | 1 |
| [^11^C]Cimbi-701 | 2 | 2 | 2 | 2 |
| [^11^C]Cimbi-717 | 3 | 2 | 2 | 2 |
| [^11^C]BA-10 | 2 | 2 | 2 | 1 |
| [^18^F]Fallypride | 2 | 2 | 2 | 2 |

**Additional file 1: Table S3:** Comparison of tracer evaluation outcomes in rats, pigs and humans.

| Tracer | rat 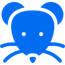 | | | | | | pig 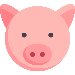 | | | | | Confirmed target-specific binding | | | Prediction outcome^d^ | human 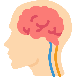 | |
| --- | --- | --- | --- | --- | --- | --- | --- | --- | --- | --- | --- | --- | --- | --- | --- | --- | --- |
|  | 60 min AUC^a^, min×g/mL | | Target-specific binding | | | | 60 min AUC^a^, min× g/mL | Target-specific binding | | | |  |  |  |  | Target-specific binding | |
|  |  |  | Std^b^ | | P-gpI^c^ | |  |  |  |  |  |  |  |  |  |  |  |
|  | Std^b^ | P-gpI^c^ | Baseline SBR | SBR change, % | Baseline SBR | SBR change, % |  | Baseline BP_ND_ | Achieved blocking effect, % | Method | Ref | rats  Std^b^ | rats  P-gpI^c^ | pigs |  | Baseline BP_ND_ range | Ref |
| [^18^F]MH.MZ | 60 | 147 | 0.71 | –97 | 1.04 | –96 | 105 | 3.3 | 81 | BP_ND_ | [9] | **Yes** | **Yes** | **Yes** | 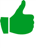 | 0.95-1.52 | [10] |
| [^18^F]Altanserin | 28 | 53 | 0.33 | +172 | 0.56 | –42 | 122 | 0.68 | 78 | BP_ND_ | [9] | *No* | **Yes** | **Yes** | 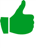 | 1.44-1.51 | [11,12] |
| [^11^C]Pimavanserin | 23 | 180 | 0.02 | +110 | 0.03 | –395 | 103 | 0.25 | n.e.^e^ | vis .ex.^e^ | [13] | *No* | *No* | *No* | 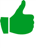 | n.d. | none |
| [^11^C]Cimbi-36 | 36 | 174 | 0.18 | –44 | 0.65 | –79 | 75 | 0.70 | 63 | BP_ND_ | [14] | **Yes** | **Yes** | **Yes** | 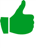 | 1.41-1.73 | [12,15] |
| [^11^C]Cimbi-717 | 43 | 62 | 0.10 | –5 | 0.14 | +46 | 155 | 6.40 | 60 | LP | [16] | *No* | *No* | **Yes** | 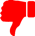 | n.d. | none |
| [^11^C]Cimbi-701 | 38 | 142 | –0.01 | –165 | 0.18 | +23 | 118 | 2.26 | 63 | LP | [17] | *No* | *No* | **Yes** | 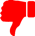 | n.d. | none |
| [^11^C]BA-10 | 29 | 65 | 0.08 | +92 | 0.16 | –44 | 107 | 0.98 | 35 | LP | this work | *No* | **Yes** | **Yes** | 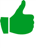 | n.d. | none |
| [^18^F]Fallypride | 95 | 244 | 3.11 | –100 | 4.07 | –95 | 191 | 9.82 | n.d.^f^ | none | this work | **Yes** | **Yes** | **Yes** | 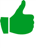 | 22-35 | [18,19] |

Scan duration in rats was 60 min for ^11^C-tracers and 90 min for ^18^F-tracers. Scan duration in pigs was 150 min for [^18^F]MH.MZ and [^18^F]Fallypride, 90 min for [^11^C]Pimavanserin, [^11^C]Cimbi-36, [^11^C]Cimbi-717, [^11^C]Cimbi-701 and [^11^C]BA-10, 60 min for [^18^F]Altanserin. Values that characterize target-specific binding are calculated from full-length scan data, while AUC values characterizing brain uptake are all truncated to the first 60 min of the scan. In pig experiments, the same drugs were used for target block as in rat experiments. Blocking effect is expressed as a decrease in BP_ND_ in the target region [20] or as an occupancy value calculated from the Lassen plot (LP) [8]. Detailed descriptions of the pig experiments can be found in the respective references and in the “Pig PET experiments” section above. BP_ND_ for [^11^C]Pimavanserin is calculated from cortical and cerebellar V_T_ values reported in [13] using Equation I (see “Pig PET experiments”). TACs for [^11^C]BA-10 and [^18^F]fallypride and Lassen plot for [^11^C]BA-10 in pigs are presented in Additional file 1: Fig. 6-8.

^a^ Average AUC values are shown for target receptor-rich regions of the brain (medial prefrontal cortex / frontal cortex / cortex for 5-HT_2A_ tracers, thalamus for 5-HT_7_ tracers, striatum for D_2/3_ tracers) ^b^ Std = standard evaluation workflow without P-gp inhibition; ^c^ P-gpI = P-gp inhibition; ^d^ Prediction outcome indicates if the evaluation outcome in pigs could (thumb up) or could not (thumb down) be correctly predicted from evaluation outcome in rats; ^e^ no effect detected by visual examination of TACs at baseline and after target block; ^f^ not determined

**Additional file 1: Table S4**. Comparison of 5-HT_2A_, 5-HT_7_ and D_2/3_ receptor densities in the brain across species.

| Receptor | Region of interest | Radioligand used to measure receptor density | Receptor density, fmol/mg tissue | | | Ref |
| --- | --- | --- | --- | --- | --- | --- |
|  |  |  | rat | pig | human |  |
| 5-HT_2A_ | frontal cortex | [^3^H]MDL100907 | 93-166 | 29±3 | 56-70 | [9,21,22] |
| 5-HT_7_ | thalamus | [^3^H]SB269970 | 47-83 | 46-62 | 9-16 | [16,23,24] |
| D_2/3_ | striatum | benzamide radioligands | 166±90 | 102±7 | 166±204 | [25] |

# References

1. Hansen HD, Lacivita E, Di Pilato P, Herth MM, Lehel S, Ettrup A, et al. Synthesis, radiolabeling and in vivo evaluation of [11C](R)-1-[4-[2-(4-methoxyphenyl)phenyl]piperazin-1-yl]-3-(2-pyrazinyloxy)-2-propanol, a potential PET radioligand for the 5-HT7 receptor. Eur J Med Chem. Elsevier; 2014;79:152–63.

2. Hong IK, Chung ST, Kim HK, Kim YB, Son YD, Cho ZH. Ultra Fast Symmetry and SIMD-Based Projection-Backprojection (SSP) Algorithm for 3-D PET Image Reconstruction. IEEE Trans Med Imaging. 2007;26:789–803.

3. Sureau FC, Reader AJ, Comtat C, Leroy C, Ribeiro M-J, Buvat I, et al. Impact of Image-Space Resolution Modeling for Studies with the High-Resolution Research Tomograph. J Nucl Med. 2008;49:1000–8.

4. Keller SH, Svarer C, Sibomana M. Attenuation Correction for the HRRT PET-Scanner Using Transmission Scatter Correction and Total Variation Regularization. IEEE Trans Med Imaging. 2013;32:1611–21.

5. Villadsen J, Hansen HD, Jørgensen LM, Keller SH, Andersen FL, Petersen IN, et al. Automatic delineation of brain regions on MRI and PET images from the pig. J Neurosci Methods. 2018;294:51–8.

6. Cumming P, Rosa-Neto P, Watanabe H, Smith D, Bender D, Clarke PB., et al. Effects of acute nicotine on hemodynamics and binding of [11C]raclopride to dopamine D2,3 receptors in pig brain. Neuroimage. 2003;19:1127–36.

7. Lammertsma A a, Hume SP. Simplified Reference Tissue Model for PET Receptor Studies. Neuroimage. 1996;4:153–8.

8. Cunningham VJ, Rabiner EA, Slifstein M, Laruelle M, Gunn RN. Measuring drug occupancy in the absence of a reference region: the Lassen plot re-visited. J Cereb Blood Flow Metab. Nature Publishing Group; 2010;30:46–50.

9. Hansen HD, Ettrup A, Herth MM, Dyssegaard A, Ratner C, Gillings N, et al. Direct comparison of [ 18 F]MH.MZ and [ 18 F]altanserin for 5-HT 2A receptor imaging with PET. Synapse. Wiley Online Library; 2013;67:328–37.

10. Kramer V, Dyssegaard A, Flores J, Soza-Ried C, Rösch F, Knudsen GM, et al. Characterization of the serotonin 2A receptor selective PET tracer (R)-[18F]MH.MZ in the human brain. Eur J Nucl Med Mol Imaging. European Journal of Nuclear Medicine and Molecular Imaging; 2020;47:355–65.

11. Sadzot B, Lemaire C, Maquet P, Salmon E, Plenevaux A, Degueldre C, et al. Serotonin 5HT2 receptor imaging in the human brain using positron emission tomography and a new radioligand, [18F]altanserin: Results in young normal controls. J Cereb Blood Flow Metab. 1995;15:787–97.

12. Ettrup A, Svarer C, McMahon B, da Cunha-Bang S, Lehel S, Møller K, et al. Serotonin 2A receptor agonist binding in the human brain with [11C]Cimbi-36: Test-retest reproducibility and head-to-head comparison with the antagonist [18F]altanserin. Neuroimage. Elsevier B.V.; 2016;130:167–74.

13. Andersen VL, Hansen HD, Herth MM, Dyssegaard A, Knudsen GM, Kristensen JL. 11C-labeling and preliminary evaluation of pimavanserin as a 5-HT2A receptor PET-radioligand. Bioorganic Med Chem Lett. 2015;25:1053–6.

14. Ettrup A, Hansen M, Santini MA, Paine J, Gillings N, Palner M, et al. Radiosynthesis and in vivo evaluation of a series of substituted 11C-phenethylamines as 5-HT2A agonist PET tracers. Eur J Nucl Med Mol Imaging. Springer; 2011;38:681–93.

15. Ettrup A, da Cunha-Bang S, McMahon B, Lehel S, Dyssegaard A, Skibsted AW, et al. Serotonin 2A receptor agonist binding in the human brain with [^11^C]Cimbi-36. J Cereb Blood Flow Metab. 2014;34:1188–96.

16. Hansen HD, Herth MM, Ettrup A, Andersen VL, Lehel S, Dyssegaard A, et al. Radiosynthesis and In Vivo Evaluation of Novel Radioligands for PET Imaging of Cerebral 5-HT7 Receptors. J Nucl Med. 2014;55:640–6.

17. L ’Estrade ET, Shalgunov V, Edgar FG, Strebl-Bantillo MG, Xiong M, Crestey F, et al. Radiosynthesis and preclinical evaluation of [ 11 C]Cimbi-701 - Towards the imaging of cerebral 5-HT 7 receptors. J Label Compd Radiopharm. 2020;63:46–55.

18. Mukherjee J, Christian BT, Dunigan KA, Shi B, Narayanan TK, Satter M, et al. Brain imaging of18F-fallypride in normal volunteers: Blood analysis, distribution, test-retest studies, and preliminary assessment of sensitivity to aging effects on dopamine D-2/D-3 receptors. Synapse. Wiley; 2002;46:170–88.

19. Vernaleken I, Fellows C, Janouschek H, Bröcheler A, Veselinovic T, Landvogt C, et al. Striatal and extrastriatal D2/D3-receptor-binding properties of ziprasidone: a positron emission tomography study with [18F]Fallypride and [11C]raclopride (D2/D3-receptor occupancy of ziprasidone). J Clin Psychopharmacol. 2008;28:608–17.

20. Innis RB, Cunningham VJ, Delforge J, Fujita M, Gjedde A, Gunn RN, et al. Consensus nomenclature for in vivo imaging of reversibly binding radioligands. J Cereb Blood Flow Metab. 2007;27:1533–9.

21. Herth MM, Knudsen GM, Herth M. M. KGM. PET Imaging of the 5-HT2A Receptor System: A Tool to Study the Receptor’s In Vivo Brain Function. In: Guiard B. DGG, editor. 5-HT2A Recept Cent Nerv Syst. Humana Press, Cham; 2018. p. 86–135.

22. Lopez-Gimenez JF, Villazon M, Brea J, Loza MI, Palacios JM, Mengod G, et al. Multiple Conformations of Native and Recombinant Human 5-Hydroxytryptamine2A Receptors Are Labeled by Agonists and Discriminated by Antagonists. Mol Pharmacol. 2001;60:690–9.

23. Horisawa T, Ishiyama T, Ono M, Ishibashi T, Taiji M. Binding of lurasidone, a novel antipsychotic, to rat 5-HT7 receptor: Analysis by [3H]SB-269970 autoradiography. Prog Neuro-Psychopharmacology Biol Psychiatry. Elsevier Inc.; 2013;40:132–7.

24. Varnäs K, Thomas DR, Tupala E, Tiihonen J, Hall H. Distribution of 5-HT7 receptors in the human brain: A preliminary autoradiographic study using [3H]SB-269970. Neurosci Lett. 2004;367:313–6.

25. Cumming P. Absolute abundances and affinity states of dopamine receptors in mammalian brain: A review. Synapse. 2011;65:892–909.
